# COVID-19 and Preeclampsia: Overlapping Features in Pregnancy

**DOI:** 10.5041/RMMJ.10464

**Published:** 2022-01-27

**Authors:** Ramasamy Sathiya, Jayanthi Rajendran, Saravanan Sumathi

**Affiliations:** Department of Biochemistry, Mahatma Gandhi Medical College & Research Institute, Sri Balaji Vidyapeeth (Deemed to be University), Puducherry, India

**Keywords:** Angiotensin-converting enzyme (ACE), COVID-19, preeclampsia, preeclampsia-like syndrome, SARS-CoV-2, sFlt-1

## Abstract

Coronavirus disease 2019 (COVID-19) is a global respiratory disease with unique features that have placed all medical professionals in an alarming situation. Preeclampsia is a hypertensive disorder of pregnancy affecting 8%–10% of India’s pregnant population. Assuming that severe acute respiratory syndrome coronavirus 2 (SARS-CoV-2) enters host cells through the angiotensin-converting enzyme 2 (ACE2) receptor, the resulting symptoms are due to vasoconstriction, caused by disturbances in the renin–angiotensin system (RAS). Other features of preeclampsia include endothelial dysfunction due to placental ischemia, leading to imbalances in angiogenic and antiangiogenic factors which result in increased blood pressure, proteinuria, altered hepatic enzymes, renal failure, and thrombocytopenia, amongst others. The increased prevalence of preeclampsia that was seen among mothers with SARS-CoV-2 infection might be due to misdiagnosis, as COVID-19 and preeclampsia have coincidental medical features. The major similarities of SARS-CoV-2-infected and preeclamptic women are a rise in pro-inflammatory cytokines, and increased serum ferritin and thrombocytopenia. Therefore, differential diagnosis might be difficult in pregnant women with COVID-19 who present with hypertension and proteinuria, thrombocytopenia, or elevated liver enzymes. The most promising markers for earlier diagnosis of preeclampsia is soluble endoglin (sEng), pregnancy-associated plasma protein-A (PAPP-A), soluble fms-like tyrosine kinase 1 (sFlt-1), and placental growth factor (PlGF). Due to placental hypoxia, sFlt-1 will be overproduced, thus inhibiting PlGF, and this alteration will be observed in the circulation five weeks or more before the onset of symptoms. The sFlt-1/PlGF ratio may also be modified via infectious states, but unregulated levels of those mediators are related to placental insufficiency. Hence, pregnant women with COVID-19 may develop a preeclampsia-like syndrome that might be differentiated properly by angiogenic markers to avoid unnecessary interventions and induced preterm labor.

## INTRODUCTION

Severe acute respiratory syndrome coronavirus 2 (SARS-CoV-2) is possibly the most notable infectious virus encountered worldwide. Due to its rapid progression and the absence of precise remedial measures, it has evolved as a global pandemic.^1^ The primary action of SARS-CoV-2 is on the lungs, followed by liver damage, thrombocytopenia, hypertension, and kidney damage.^2^ Transmission is through droplets and close contact, and the virus can particularly affect individuals with weakened immune systems, including pregnant women, senior citizens, and patients with other comorbidities.^3^ Furthermore, the severity of SARS-CoV-2 infections can range from asymptomatic infection to serious respiratory failure.^4^

Pregnant women could be more susceptible to SARS-CoV-2 due to the immunological and physiological adaptive remodeling that occurs during pregnancy.^5^ Furthermore, SARS-CoV-2 also causes hypoxic injury to the placenta, which could contribute to the development of preeclampsia.^6^ Another factor making pregnant women more vulnerable to coronavirus disease 2019 (COVID-19) is that the virus enters the cells by means of the receptor protein angiotensin-converting enzyme 2 (ACE2). By converting angiotensin (Ang) I and Ang II into Ang-(1-9) and Ang-(1-7), respectively, ACE2 regulates the renin–angiotensin system (RAS). Upregulation of ACE2 occurs in the placenta and fetus during pregnancy, making the placenta a potential site for SARS-CoV-2 infection and vertical transmission to the fetus.^7^,^8^ Studies are showing that pregnant woman infected with SARS-CoV-2 are at higher risk of preterm delivery, low birth weight, and spontaneous abortion.^9^,^10^ Furthermore, a systematic review conducted by Ciapponi et al. has reported that antenatal mothers infected with this virus are more prone for preeclampsia, cesarean birth, miscarriage, and perinatal death.^11^

Preeclampsia is a hypertensive disorder of pregnancy that affects nearly 5% of pregnancies worldwide and is one of the leading causes of maternal and fetal morbidity and mortality. It is characterized by new onset of hypertension after 20 weeks of gestation, with or without proteinuria ≥300 mg/24 hours urine collection. The actual cause of preeclampsia remains unknown, and the only curative action is placental removal.^12^ Risk factors for preeclampsia include primigravida, pre-pregnancy weight, age, previous and family history of preeclampsia, and life style modifications during pregnancy. Pregnant women aged <16 years or more than >40 years are more prone for the disease.^13^ Generally, preeclampsia is a combination of proteinuria and hypertension. However, if hemolysis, elevated liver enzymes, low platelet (HELLP) syndrome is present, the result can be a more severe form of preeclampsia.^14^

The presence of HELLP syndrome is the classical feature in high-risk cases of preeclampsia.^15^ However, abnormal liver enzymes are present not only during pregnancy, but also in critically ill patients^16^ and in certain infectious diseases.^17^,^18^ Different studies have stressed that viral diseases, and notably COVID-19, mimic HELLP syndrome.^16^,^19^,^20^ This raises the question as to whether or not misdiagnoses have occurred in a number of cases, since SARS-CoV-2 infection and preeclampsia have overlapping clinical features, making differential diagnosis difficult in SARS-CoV-2-infected antenatal women presenting with proteinuria, hypertension, thrombocytopenia, and altered liver enzymes.^21^ This review describes the clinical data of SARS-CoV-2-positive subjects and preeclamptic women based on laboratory findings.

## PATHOPHYSIOLOGY OF PREECLAMPSIA

As a placental disease, preeclampsia progresses at two different levels: (1) Abnormal placentation is noted during the early stage of pregnancy; (2) maternal syndrome occurs after 20 weeks’ gestation and is distinguished by the secretion of excess antiangiogenic factors in the blood.^22^ During normal placentation, three major changes occur. First, the uterine lining transforms into a dense cellular matrix known as the decidua. Second, the fetal trophoblast penetrates deep into the myometrium of the maternal decidua, followed by invasion of maternal spiral arteries that replace the endothelial and other smooth muscle cells. Finally, the spiral arteries differentiate into huge low-resistance vessels, resulting in the extended maternal flow to the placenta.^23^ If something goes wrong during the above process, the result will be improper placentation, placental hypoxia, and poor invasion of spiral arteries, culminating in the release of vasoactive factors into the circulation. Biomarkers for early identification of preeclampsia are critical for threat stratification and treatment modalities. Potential preeclampsia biomarkers can be grouped into the following categories^24^: reactive oxygen species, genetics, placental or trophoblast ischemia/hypoxia, vascular endothelial dysfunction, and immune maladaptation.^25^

### Role of Biomarkers

Biomarkers are becoming increasingly significant in the diagnosis of preeclampsia. A survey of the literature reveals a number of biomarkers that have been shown to be sufficiently specific and sensitive to qualify as possible biomarkers. An effective biomarker is one that has the ability to detect preeclamptic women earlier in their disease progression. Much research has also looked into various systems for predicting preeclampsia.

#### Soluble fms-like tyrosine kinase-1 (sFlt-1) and placental growth factor (PlGF)

Soluble fms-like tyrosine kinase-1 (sFlt-1) is the splice variant of vascular endothelial growth factor (VEGF) receptor-1, which is secreted in response to placental hypoxia; once sFlt-1 binds to VEGF in the extracellular domain, VEGF becomes soluble in plasma. Because sFlt-1 has the VEGF binding site, it can bind all isoforms of the growth factor, as well as proangiogenic protein PlGF.^22^,^26^ In healthy pregnancies, after 30–32 weeks of gestation, the amount of sFlt-1 begins to increase, and the level of PlGF begins to drop, mainly due to cellular tension in the syncytiotrophoblast during the last 8–10 weeks of pregnancy, leading to alterations in sFlt-1 and PlGF levels. In preeclamptic patients, circulating levels of sFlt-1 and PlGF vary. This change happens before the manifestation of symptoms and continues throughout the course of the illness. Levels of sFlt-1 rise roughly 5 weeks before manifestation of symptoms in women with preeclampsia, but PlGF levels fall before sFlt-1 levels rise.^27^ Hence, screening pregnant women during the first trimester to identify those at risk for preeclampsia could enable the use of low-dose aspirin to prevent maternal and neonatal complications.^22^

#### Soluble endoglin (sEng)

Soluble endoglin is a putative antiangiogenic factor that influences the production of nitric oxide (NO), vasodilation, and capillary formation by endothelial cells *in vitro* by interfering with transforming growth factor 1 (TGF1) binding to its receptor.

Soluble endoglin can produce high blood pressure by affecting the permeability of blood arteries *in vivo*, and it can also block capillary angiogenesis *in vitro*. Serum sEng may rise during the last two months of pregnancy in a normal pregnancy, but it rapidly increases in preeclampsia, surging at onset, and it may be linked to disease severity. Further-more, sEng may work in concert with sFlt-1 in the etiology of preeclampsia. As a result, it is believed that elevated sEng levels in the blood and a higher sFlt-1:PlGF ratio can predict the likelihood of preeclampsia,^28^ but more clinical evidence is needed to confirm this hypothesis.

#### Pregnancy-associated plasma protein-A

Pregnancy-associated plasma protein-A (PAPP-A) is a glycoprotein produced primarily in placental trophoblast cells. Its presence could indicate placental ischemia or hypoxia. Essential for a normal pregnancy, PAPP-A can alter placental trophoblast cell infiltration by regulating the action of insulin-like growth factors. Placental PAPP-A is often used in aneuploidy screening in early pregnancy, and it can be used to detect intrauterine growth retardation (IUGR), whereas it cannot detect preeclampsia.^29^

### Inflammatory Markers

Although pregnancy is a “healthy” physiological process, the fetus may pose a risk to maternal tolerance and the immune system. A healthy pregnancy necessitates maternal immune system tolerance of the semi-allogenic fetus, and the fetus must be safeguarded against rejection by the maternal immunological response.^30^ An ischemic placenta may increase inflammatory cytokine production in preeclampsia. Increased pro-inflammatory cytokines and decreased anti-inflammatory cytokines are produced by an imbalance in CD4+T cells in preeclampsia. Interleukin (IL)-6 may disrupt the equilibrium of CD4+T cells during placental ischemia. The T helper-17 cells, a subclass of CD4+T cells, are responsible for the secretion of inflammatory cytokine IL-17, which is elevated in preeclampsia.^28^

### Reactive Oxygen Species

Reactive oxygen species (ROS) are oxygen-containing highly reactive compounds produced during aerobic cell metabolism. During pregnancy there is an improved production of ROS, but it is commonly counterbalanced by antioxidants. In preeclampsia, faulty trophoblast invasion and diminished uteroplacental blood flow lead to intervals of ischemia/reperfusion and the hypoxic surroundings that favor oxidative stress, inflammation, and vascular dysfunction. The antioxidant levels during preeclampsia can be too low to counterbalance the increased ROS. Neutrophils and monocytes are key sources of ROS in preeclampsia. Monocytes from preeclamptic women increase H_2_O_2_ and O_2_^•−^, resulting in greater endothelial dysfunction as compared to monocytes produced from normotensive pregnant women.^13^

## CLINICAL PRESENTATION OF PREECLAMPSIA

The incidence of preeclampsia is 3%–7% in nulliparous pregnancies. One of the leading causes of maternal morbidity and mortality,^12^ it is strongly associated with negative outcomes such as placental abruption, spontaneous abortion, stillbirth, intrauterine growth retardation, and preterm labor.^31^ Clinical and laboratory tests are used to diagnose, follow, and regulate the severity of preeclampsia. Increased blood pressure, headaches, and blurring of the vision may all be associated with cerebral edema; and the presence of HELLP syndrome may be detected by oliguria, vaginal bleeding, epigastric pain, and vomiting. Laboratory tests for diagnoses will include 24 h urinary protein to detect proteinuria; a complete blood count, including platelet count, to detect thrombocytopenia; bilirubin, alanine amino transferase (ALT), and aspartate amino transferase (AST) levels to detect the presence of HELLP syndrome; and serum electrolytes, urea, and creatinine to check for acute renal failure (see also Table 1). Fetal investigations are also used, including Doppler ultrasound to determine the uterine artery pulsability index, scrutiny of the placenta, fetal weight, and fetal well-being based on the Manning score.^15^

**Table 1:** Comparison of SARS-CoV-2-Positive Pregnant Women and Preeclamptic Women.

Characteristics	SARS-CoV-2-Positive Pregnant Women	Preeclamptic Women	Reference
Pro-inflammatory cytokines	Interleukin (IL)-2, IL-6, IL-7, and tumor necrosis factor-α (TNFα)^23^	IL-6, IL-10, and TNFα^6^	Martínez-Varea et al.^23^ Abbas et al.^6^
Serum ferritin	Increased^6^	Increased^6^	Abbas et al.^6^
Platelets	Thrombocytopenia: Defining criteria for cytopenia in H-score^32^	Thrombocytopenia^33^: Independent risk factor	Reddy and Rajendra Prasad^34^
AST, ALT, LDH	Increased^2^	Increased^33^	Pereira et al.^2^ Hassanpour et al.^33^
Total bilirubin	Increased^35^	Increased^33^	Agarwal et al.^35^ Hassanpour et al.^33^

ALD, alanine amino transferase; AST, aspartate amino transferase; LDH, lactate dehydrogenase.

## SARS-CoV-2 INFECTION AND PREGNANCY

### Angiotensin-converting Enzyme (ACE) during Pregnancy

**Figure 1:**
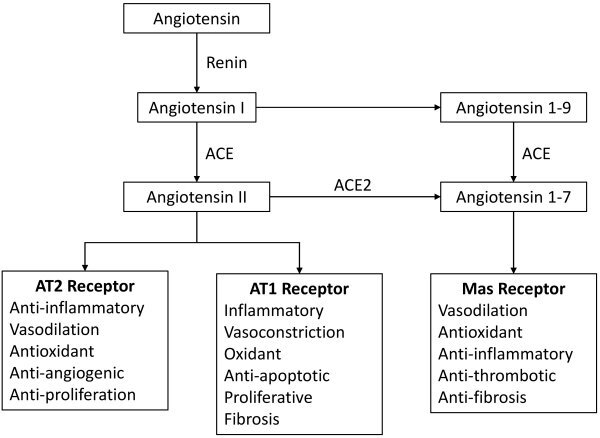
Schematic Representation of Renin-Angiotensin Axis. ACE, angiotensin-converting enzyme; ACE2, angiotensin-converting enzyme 2; AT2 receptor, angiotensin type II receptor; AT1 receptor, angiotensin type I receptor.

Structural analyses have shown that SARS-CoV-2 shares 79% of the sequence found in severe acute respiratory syndrome coronavirus (SARS-CoV) and that they may both have the same host receptor, ACE2.^36^ Furthermore, COVID-19 also seems to have features that can impair maternal and fetal well-being. The RAS is primarily regulated by ACE2, which converts Ang I and Ang II into Ang-(1-9) and Ang-(1-7), respectively. A powerful vasopressor response occurs when Ang II binds to AT1 receptors, which are numerous and broadly distributed in hu-man tissues. By activating the AT1 receptors, Ang II exerts proinflammatory, prooxidant, proangiogenic, and antiapoptotic actions. Finally, Ang II increases aldosterone and vasopressin release by the adrenal glands and the neurohypophysis, facilitating sodium and water reabsorption in the kidneys. Vasodilation, apoptosis, antioxidant defenses, and antiproliferative and antiangiogenic responses stimulated by AT2 are the same as for AT1 stimulation. On the other hand, AT2 is less abundant in adult tissues, being expressed more in the reproductive organs and fetal tissues.^37^ Angiotensin-(1-7), the primary ACE2 product, is an antagonist of the harmful effects of Ang II and AT1. When ACE2 cleaves to Ang II, Ang-(1-7) is produced. Ang-(1-7) binds to Mas, the G-protein coupled receptor, thereby promoting vasodilation and anti-inflammatory, anti-remodeling, anti-arrhythmic, and anti-proliferative actions. As a result, the ACE2/Ang-(1-7)/Mas receptor axis has attracted attention as a RAS counter-regulatory axis (Figure 1).^38^

Placentation is a complicated process that involves fetal cells, alterations in maternal uterine circulation, and immunomodulation. Most RAS components, as well as prostaglandins and nitric oxide, have been identified in the human uteroplacental unit, implying that these systems are complexly regulated throughout placental development.^39^ Both ACE2 and Ang-(1-7) are expressed by syncytiotrophoblasts, cytotrophoblasts, endothelial cells of villous blood vessels, vascular smooth muscle cells of primary villi, the syncytioblast cells, and the decidua. Prorenin, (pro)renin receptor, and AT1 and AT2 proteins are expressed in the extravillous trophoblasts, indicating that they play a vital role in trophoblast migration.^40^

The SARS-CoV-2 virus enters host cells through the membrane-bound receptor of ACE2 and CD147/basigin (BSG). Following receptor binding, the viral-encoded S protein relies on host proteases for cleavage to attain effective membrane fusion. The primary host protease that mediates S protein priming and initiates viral access is the type II transmembrane serine protease, TMPRSS2.^41^

In normal pregnancy the concentration of plasma Ang-(1-7) increases, whereas the ACE concentration decreases; this is reversed in women with preeclampsia. An increased ACE concentration was also reported in women who delivered small-for-gestational-age babies. As placental cells express increased concentrations of ACE2, TMPRSS2 provides an increased affinity to the spike protein.^41^

### Biochemical Features of Pregnant Women Infected with SARS-CoV-2

Studies that examined maternal deaths in SARS-CoV-2-positive antenatal women showed increased AST, ALT,^42^,^43^ total bilirubin, cardiac enzymes, serum creatinine, and urea.^44^ The same enzymatic changes can also occur in preeclamptic women. Angiotensin-converting enzyme 2 has varying tissue localization (lung, placenta, liver, kidney, brain, etc.); it is also a major regulator of blood pressure and additionally acts as a functional receptor for SARS-CoV-2 too.^45^ Therefore, an increase in hepatic enzymes in pregnancy could be due to ACE2 binding with SARS-CoV-2, upregulating ACE2 expression in both liver cells and cholangiocytes^35^ in SARS-CoV-2 patients, but the actual mechanism behind the scenario is unclear.

The major similarities between SARS-CoV-2-infected antenatal women and preeclamptic women are increased pro-inflammatory cytokines and serum ferritin, and thrombocytopenia (Table 1).^6^ In typical pregnancies, successful invasion is predicated upon an appropriate interface between trophoblast cells and maternal epithelial, immune, and endothelial cells and tissues. Hence, the maternal immune cells play a crucial role in assisting the interaction between two immunologically distinctive beings.^46^

The differential diagnosis of preeclampsia during pregnancy from SARS-CoV-2-positive pregnant women is via assessment of the placental vascular development through VEGF, PlGF, and antiangiogenic factor sFlt-1. Dysregulation of this balance can also happen due to inflammatory mediators, including cytokines (e.g. IL-1, INF-γ, TNF) and the complement cascade pathway. The sFlt-1/PlGF ratio may also be modified as the result of infectious states, but unregulated levels of those mediators are related to placental insufficiency, placental hypoxia, and poor nutrient transport to the fetus, which lead to poor neonatal outcome.^44^

Mendoza et al. conducted a study on 42 SARS-CoV-2-positive pregnant women; five of the subjects developed clinical and laboratory symptoms similar to preeclampsia. Of the five, only one was confirmed to actually have preeclampsia, presenting with sFlt-1 and PlGF, elevated platelet distribution, and increased lactate dehydrogenase (LDH). The remaining four pregnant women recovered after starting antibiotic therapy and the preeclamptic symptoms were eradicated, whereas in true preeclampsia delivery is the only curative treatment.^21^ Another study conducted by Hansen et al. reported that a 34-week gestational mother presented with hypertension and SARS-CoV-2 infection. Her blood pressure was 162/86 mmHg, laboratory parameters were altered, and transabdominal ultrasound showed normal fetal movement. The woman suddenly developed superimposed preeclampsia and was started on 10 mg of labetalol and 4 g of magnesium sulfate (MgSO_4_) intravenously. Due to elevated blood pressure and hepatic enzymes she underwent an uncomplicated cesarean delivery.^9^

## MATERNAL AND FETAL OUTCOME OF PREGNANT WOMEN SUFFERING FROM COVID-19

**Figure 2:**
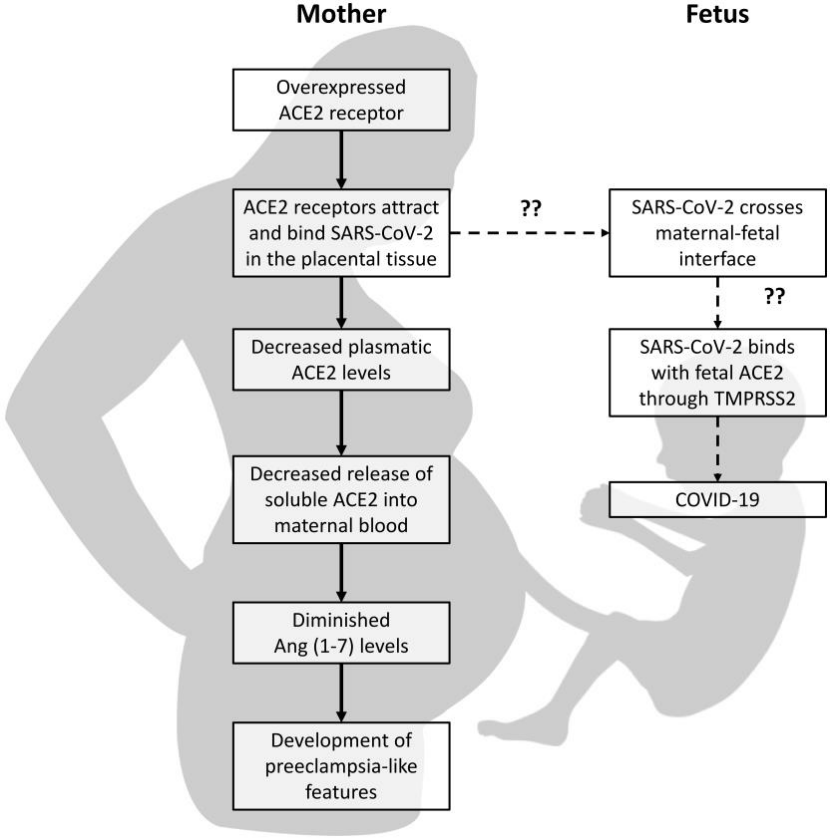
Proposed Mechanism by Which SARS-CoV-2 Symptoms Develop both in Mother and Fetus. ***Hypothesis:*** The highly expressed ACE2 receptor serves as the binding site for SARS-CoV-2, which may cause preeclampsia-like syndrome in pregnant women. For the duration of pregnancy, fetal ACE2 is overexpressed; therefore, if SARS-CoV-2 crosses the placenta and engages with fetal ACE2, the virus may induce fetal morbidity and mortality. The solid arrows indicate known mechanisms; the dashed arrows indicate the hypothesized mechanism. ??, assumed or not yet confirmed; ACE2, angiotensin-converting enzyme 2; Ang, angiotensin; SARS-CoV-2, severe acute respiratory syndrome coronavirus 2; TMPRSS2, transmembrane protease serine 2.

The impact of COVID-19 on pregnancy has recently received much attention. However, some data are contradictory and methodological flaws have been noted, such as testing for COVID-19 only in symptomatic pregnancies, a lack of control for confounding factors (such as age, comorbidities, multiparity, etc.), limited comparisons with non-pregnant women, and an under-representation of COVID-19-positive pregnancies in early pregnancy.^47^ More studies are emerging that investigate maternal and placental ACE2 expression and activity changes, as well as potential mechanisms relating ACE2 to the pathophysiology of COVID-19, since many clinicians are seeing an increased number of COVID-19 pregnancies and an association with the severity of COVID-19.^11^,^48^–^50^ The possibility that ACE2 is acting as a viral entry door to the placenta and fetal circulation, thereby boosting or enhancing intrauterine vertical transmission, is of great concern. However, a secondary effect of SARS-CoV-2 on feto–maternal circulation and placentation caused by substantial ACE2 depletion has received little attention.^6^,^43^,^48^ Based on the above theory, we hypothesize that, during pregnancy, the ACE2 receptor serves as the binding site for SARS-CoV-2, thereby augmenting the severity of COVID-19 and the incidence of unfavorable outcomes, such as premature birth and preeclampsia (Figure 2). Other COVID-19-related problems that have been observed include small-for-gestational age, IUGR, hospitalization to the neonatal intensive care unit, and stillbirth.^6^

Decreased ACE2 protein has also been reported in placentas from COVID-19-positive pregnancies, indicating that SARS-CoV-2 infection may alter ACE2 expression and biological functions in the placenta, as well as in the maternal and fetal circulations, either directly or indirectly. Vertical transmission may occur through intrauterine, intrapartum, and postpartum mechanisms. Thankfully, the majority of viral illnesses affecting mothers are not vertically transmissible.^43^,^49^ However, even viral infections that only affect the placenta can lead to functional damage, with negative consequences such as miscarriages, IUGR, and preterm birth. Initially, there was no evidence that SARS-CoV-2 was vertically transmitted; however, new reports of newborn infection are showing vertical transmission as the virus spreads globally.^36^,^50^

The following are the most common features noted in SARS-CoV-2-infected pregnant women^8^,^37^:

- The most commonly reported symptoms are fever (34%–84%), cough (28%–71%), dyspnea (18%–40%), and myalgia (16%–38%).
- Infected pregnant women were less prone to have fever, myalgia, or dyspnea than infected non-pregnant women. However, in infected pregnant women, fever and shortness of breath of any duration were linked to an elevated risk of severe maternal–fetal abnormalities.
- COVID-19 was found to have a modest maternal mortality rate (1%). However, when compared to uninfected pregnant women, the risk of maternal death increased in the presence of SARS-CoV-2 infection.
- Odds ratios (OR) of preeclampsia (OR 1.21, 95% CI 1.11–1.33) and premature birth (OR 1.17, 95% CI 1.06–1.29) were significantly greater in SARS-CoV-2-positive than-negative pregnancies.
- Mortality rates differ between countries, with Brazil having a mortality rate during May 2020 of 36/100,000 population among pregnant women with COVID-19 and 252/100,000 population of SARS-infected pregnant women. This may be explained by the high prevalence of hypertensive illnesses, particularly preeclampsia and obesity in pregnant women, as well as socioeconomic concerns and healthcare system flaws.
- Pregnancy complication: preterm birth (9%–39%). Having COVID-19 enhanced the chance of both medically recommended and spontaneous preterm birth. Even though the majority of preterm births were medically guided, around 6% were spontaneous.
- Fetal problems were observed in 0.5% of pregnant women with COVID-19, which included admission to a neonatal intensive care unit (15%), IUGR (9%), fetal distress (8.8%), miscarriage (2%), stillbirth (0.7%), and newborn mortality (0.7%). Indeed, recent meta-analyses evaluating similar outcomes in pregnancies with and without COVID-19 found that the disease increased the chance of newborn intensive care unit admission, stillbirth, and low birth weight.

## CONCLUSION

The characteristics of SARS-CoV-2-infected pregnant women and preeclamptic women seem to be more or less similar with regard to proteinuria, elevated liver enzymes, thrombocytopenia, and increased pro-inflammatory markers. Hence, diagnosis of preeclampsia could be quite difficult. In view of the above scenario, verification of VEGF, PlGF, sFlt-1, and sFlt-1/PlGF, along with other biochemical markers, plays a crucial role in the differential diagnosis of preeclampsia, and would help to avoid unnecessary interventions and induced preterm labor among SARS-CoV-2-positive pregnant women.

Based on current knowledge, placentas are more vulnerable, placing the fetus at higher risk of SARS-CoV-2 infection due to vertical transmission. Placental ACE2, which is known to be down-regulated in SARS-CoV-2 infection, may contribute to changes in important physiological processes during placental development and vascularization. This is confirmed by the fact that SARS-CoV-2 infection during pregnancy can cause a preeclampsia-like condition. The gestational age of exposure, on the other hand, may play a role in influencing placental vascular responses or consequences. Although some placentas lack co-localization between ACE2 and the transmembrane serine protease 2 (TMPRSS2)—a protease that is equally required for SARS-CoV-2 entry and cell proliferation—vertical transmission is still a topic of controversy.

## Abbreviations

ACE: angiotensin-converting enzyme
ACE2: angiotensin-converting enzyme 2
ALT: alanine amino transferase
Ang I: angiotensin I
Ang II: angiotensin II
AST: aspartate amino transferase
BSG: basigin
COVID-19: coronavirus disease 2019
HELLP: hemolysis, elevated liver enzymes, and low platelet
IL: interleukin
INF-γ: interferon gamma
LDH: lactate dehydrogenase
PlGF: placental growth factor
RAS: renin–angiotensin system
SARS-CoV-2: severe acute respiratory syndrome coronavirus 2
sEng: soluble endoglin
sFlt-1: soluble fms-like tyrosine kinase-1
TMPRSS2: transmembrane protease serine 2
TNF-α: tumor necrosis factor alpha
VEGF: vascular endothelial growth factor
